# Shape-shifting leaves depend on SPL10

**DOI:** 10.1093/plcell/koad046

**Published:** 2023-02-16

**Authors:** Carlisle Bascom

**Affiliations:** Assistant Features Editor, The Plant Cell, American Society of Plant Biologists, USA; Department of Cell and Developmental Biology, University of California San Diego, La Jolla 92093, USA

In multicellular organisms, individual cells must coordinate growth and proliferation to form a complex 3D organ. How cells coordinate growth as well as proliferation to form complex organ shapes is an on-going area of research. Confounding the question of how cells grow together to develop complex leaf structure is heteroblasty. Heteroblastic plants, including the model Arabidopsis (*Arabidopsis thaliana*), generate morphologically distinct leaves throughout their lifecycle. In this issue of *The Plant Cell*, **Hong-Bo Tang and colleagues (Tang et al. 2023)** used confocal microscopy, cell morphology quantification, forward and reverse genetics, and computer simulations to generate a model connecting cell fate to leaf shape through Arabidopsis maturation.

To develop a baseline for growth dynamics, the authors measured cell growth and leaf morphology throughout development (see Fig. 1). The first true leaf in Arabidopsis is round. When the authors measured morphological parameters at the tip and base of the first leaf, they found the first leaf had uniformly lobed cells at 9 DAI (days after initiation), indicative of complete epidermal maturation. In contrast, the seventh leaf had morphologically distinct cells at the base compared to the tip at 9 DAI, suggesting that maturation was not yet complete. In addition, the seventh leaf showed increased levels of endoreduplication as well as long cells reminiscent of so-called giant cells seen in sepals. The authors sought to elucidate a molecular mechanism for these observations.

**Figure 1.**
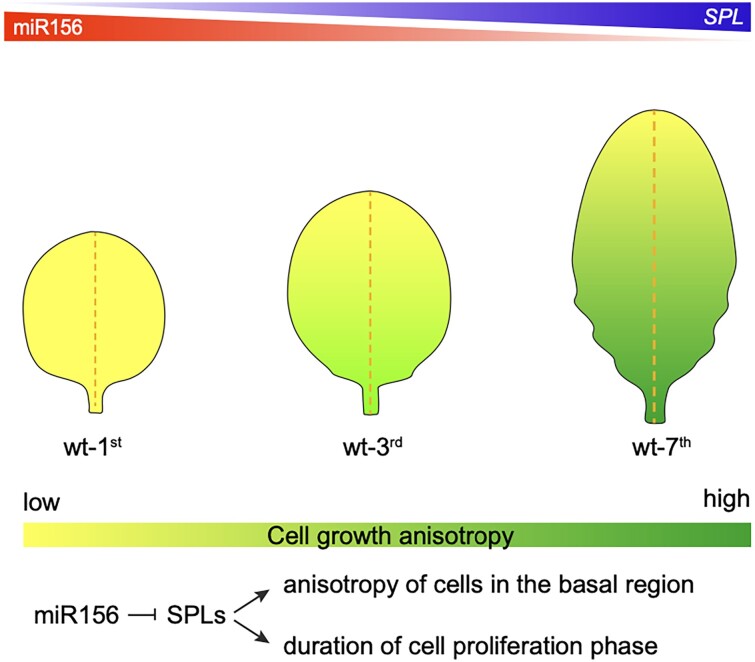
A molecular model for heteroblasty in Arabidopsis. Levels of miR156 decrease in age, resulting in an increase in SPL levels. SPLs, in turn, promote anisotropic growth of individual cells. Adapted from Tang et al. (2023), Fig. 9.

Previous reports showed that the microRNA156 (miR156) targets members of the *SQUAMOSA PROMOTER BINDING PROTEIN-LIKE* (*SPL*) transcription factor gene family, including *SPL10* (Xing et al. 2010). The reduction of *miR156* (Gao et al. 2022) or over-expression of *SPL10* (Shikata et al. 2009) results in plants that produce adult leaves earlier than wild-type. Tang et al. (2023) generated plants expressing a miR156-resistant form of *SPL10* (*rSPL10*). Observations of cell morphology over time on these plants showed that the first leaf developed much like the seventh leaf of wild-type plants. A dexamethasone-inducible form of *rSPL10* resulted in dosage-dependent morphological changes in the first leaf. Together, the authors concluded that miR156*-*regulated levels of *SPL10* are sufficient to regulate leaf shape (see Fig. 1).

Given that SPL10 is a transcription factor, the authors asked what genes are under its direct control. Using chromatin immunoprecipitation sequencing, the authors found that SPL10 binds to the promoters of genes encoding cell wall modifying proteins (xyloglucan endotransglucosylases/hydrolases and an expansin). Additionally, they found that SPL10 binds to the promoters of members of the *CYCLIN* gene family, suggesting that it is a regulator of cell proliferation. These findings made it possible to build a mathematical model whereby levels of miR156 predict leaf shape. In fact, leaf measurements of a given miR156 expression level from the model agree perfectly with those from *mir156* mutants.

Taken together, Tang and colleagues were able to propose an elegant and feasible model by which Arabidopsis accomplishes leaf heteroblasty. Moving forward, it will be exciting to assess if this mechanism of heteroblasty is conserved across the plant lineage.

## References

[koad046-B1] Gao J , Zhang K, Cheng Y-J, Yu S, Shang G-D, Wang F-X, Wu L-Y, Xu Z-G, Mai Y-X, Zhao X-Y, et al A robust mechanism for resetting juvenility during each generation in Arabidopsis. Nat Plants. 2022:8(3):257–268. 10.1038/s41477-022-01110-4 35318444

[koad046-B2] Shikata M , Koyama T, Mitsuda N, Ohme-Takagi M. Arabidopsis SBP-box genes SPL10, SPL11 and SPL2 control morphological change in association with shoot maturation in the reproductive phase. Plant Cell Physiol. 2009:50(12):2133–2145. 10.1093/pcp/pcp148 19880401

[koad046-B3] Tang H-B , Wang J, Wang L, Shang G-D, Xu Z-G, Mai Y-X, Liu Y-T, Zhang T-Q, Wang J-W. Anisotropic cell growth at the leaf base promotes age-related changes in leaf shape in *Arabidopsis thaliana*. Plant Cell. 2023:35(5):1386–1407. doi:10.1093/plcell/koad031 PMC10118278 36748203

[koad046-B4] Xing S , Salinas M, Höhmann S, Berndtgen R, Huijser P. miR156-targeted and nontargeted SBP-box transcription factors act in concert to secure male fertility in Arabidopsis. Plant Cell. 2010:22(12):3935–3950. 10.1105/tpc.110.079343 21177480 PMC3027167

